# Supplementary material for: Release of P-TEFb from the Super Elongation Complex promotes HIV-1 latency reversal
Source: bioRxiv. 2024 Mar 1:2024.03.01.582881. Preprint. [Version 1] doi: 10.1101/2024.03.01.582881 (PMC10925308; doi:10.1101/2024.03.01.582881)
Supplement: Supplement 1 — Supplemental Figure 1 | Primary cell viability and gating strategy. A) Percent viable primary CD4+ T cells (normalized to the donor-matched NT control) 72 hours after electroporation with multiplexed CRISPR-Cas9 RNPs targeting the indicated genes as measured by amine dye staining and flow cytometry. Each dot represents the average of technical triplicates; the black line represents the mean of means ± standard error. n = 12 donors for NT, CXCR4, CCNT1, CDK9, AFF1, AFF4, and ELL2; n=6 donors for AF9; n=9 donors for ENL. Statistics were calculated by 2-way ANOVA with Dunnet’s Multiple Comparison Test; no significant differences were observed. B) Gating strategy for quantification of percent viable primary CD4+ T cells via sequential application of a live cell gate, two single-cell gates, and a fluorophore gate (FlowJo v10.7.1). C) Gating strategy for quantification of percent HIV-1 infection in primary CD4+ T cells via sequential application of a live cell gate, two single-cell gates, and autofluorescence exclusion (FlowJo v10.7.1). Supplemental Figure 2 | Histograms of CD4 and CXCR4 expression on primary CD4+ T cells after KL-2 treatment. A) Histogram of cell surface CD4 expression on activated CD4+ T cells treated with DMSO or 3.125 μM KL-2 for 48 hours as measured by immunostaining and flow cytometry (one representative donor, visualized in FlowJo v10.7.1). B) Histogram of cell surface CXCR4 expression on activated CD4+ T cells treated with DMSO or 3.125 μM KL-2 for 48 hours as measured by immunostaining and flow cytometry (one representative donor, visualized in FlowJo v10.7.1). Supplemental Figure 3 | J-Lat cell viability upon LRA treatment and reactivation. A) Percent viable J-Lat 5A8 cells (normalized to the DMSO treated control) 48 hours after treatment with the indicated LRAs in the presence and absence of 6.25 μM KL-2 as measured by amine dye staining and flow cytometry. Each bar represents the average ± standard deviation of technical triplicates. The same [file NIHPP2024.03.01.582881v1-supplement-1.pdf]

Supplemental Figure 1

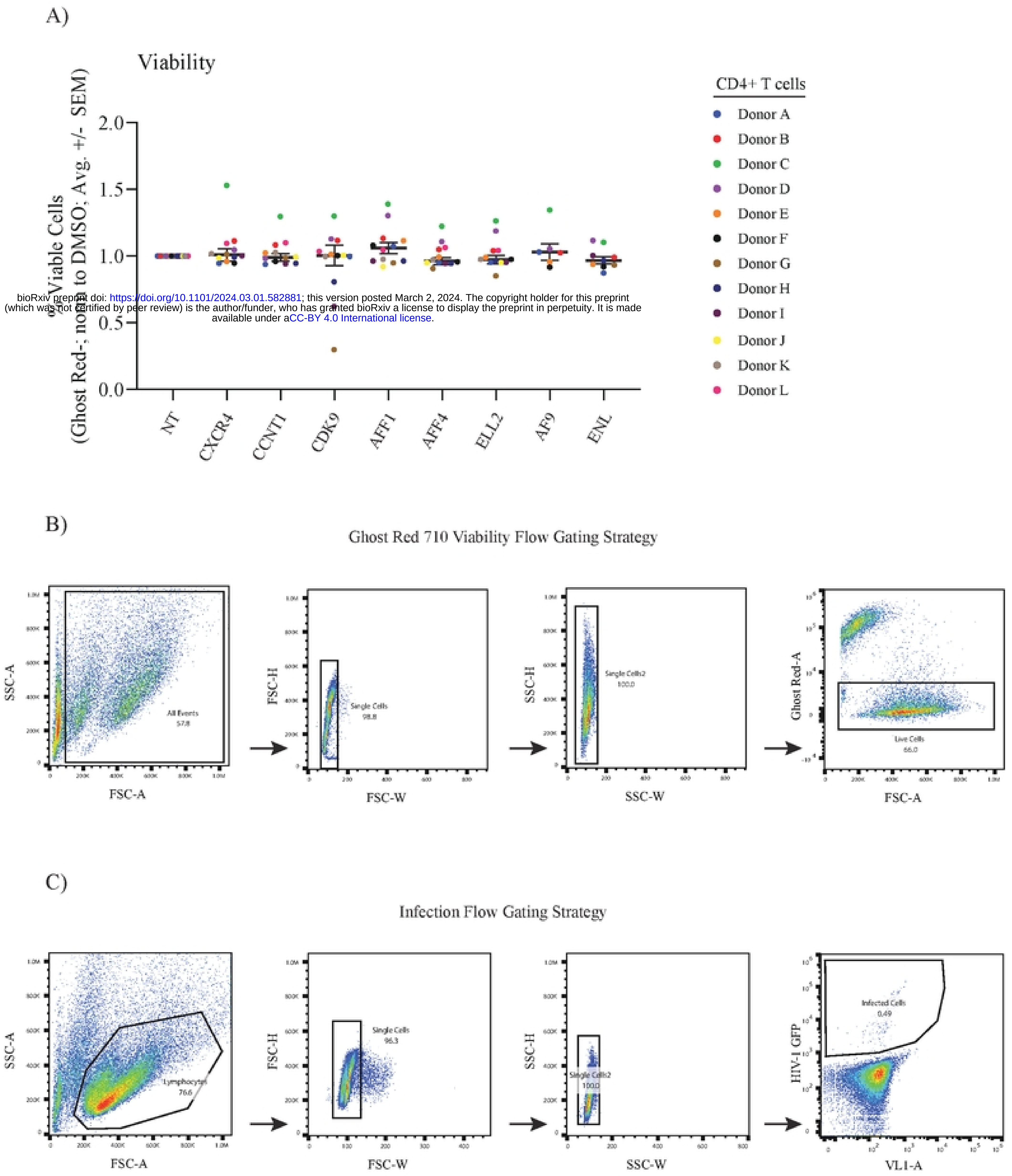

bioRxiv preprint doi: https://doi.org/10.1101/2024.03.01.582881; this version posted March 2, 2024. The copyright holder for this preprint (which was not certified by peer review) is the author/funder, who has granted bioRxiv a license to display the preprint in perpetuity. It is made available under aCC-BY 4.0 International license.

Supplemental Figure 2

bioRxiv preprint doi: https://doi.org/10.1101/2024.03.01.582881; this version posted March 2, 2024. The copyright holder for this preprint (which was not certified by peer review) is the author/funder, who has granted bioRxiv a license to display the preprint in perpetuity. It is made available under aCC-BY 4.0 International license.

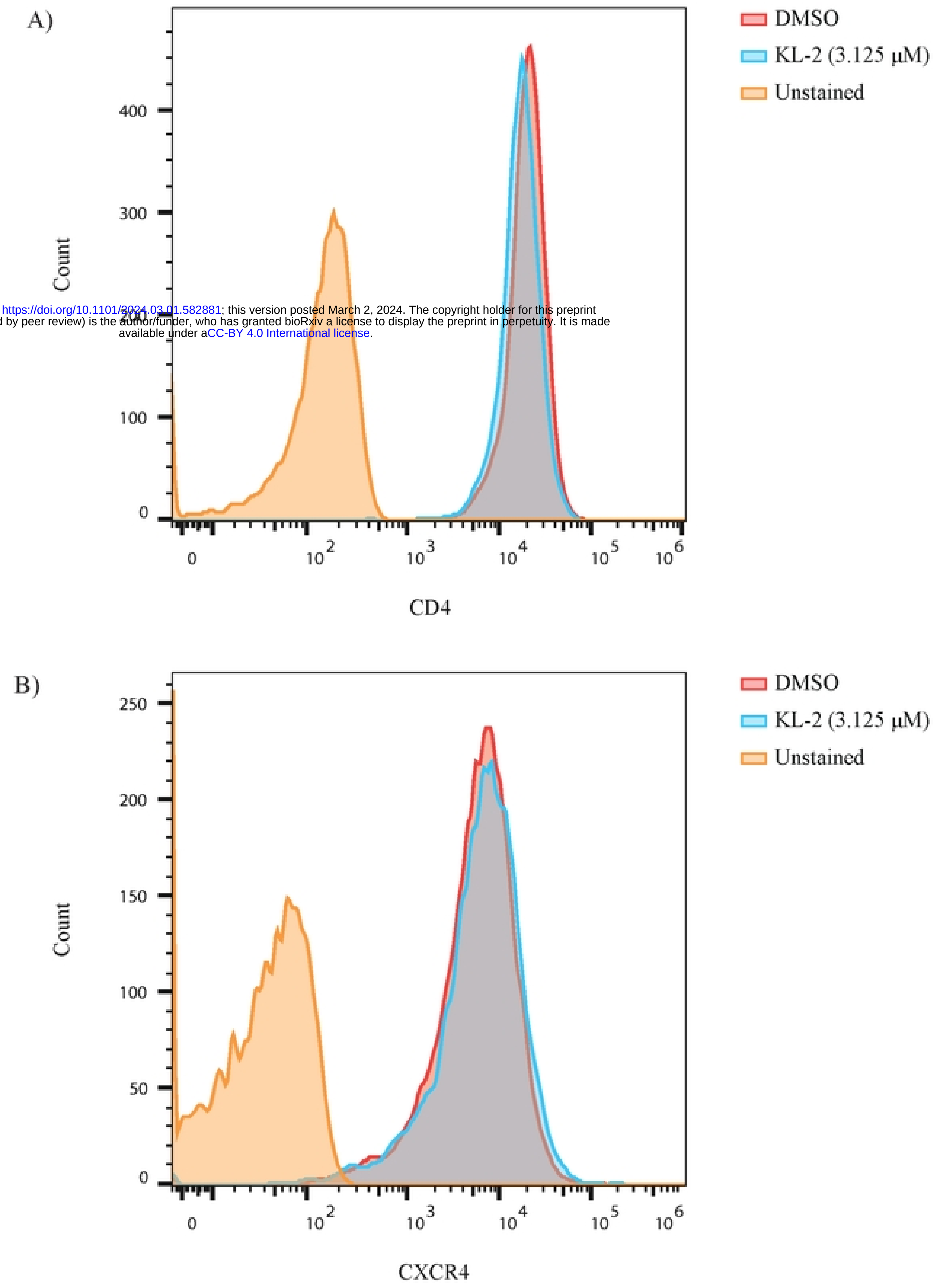

Supplemental Figure 3

A)

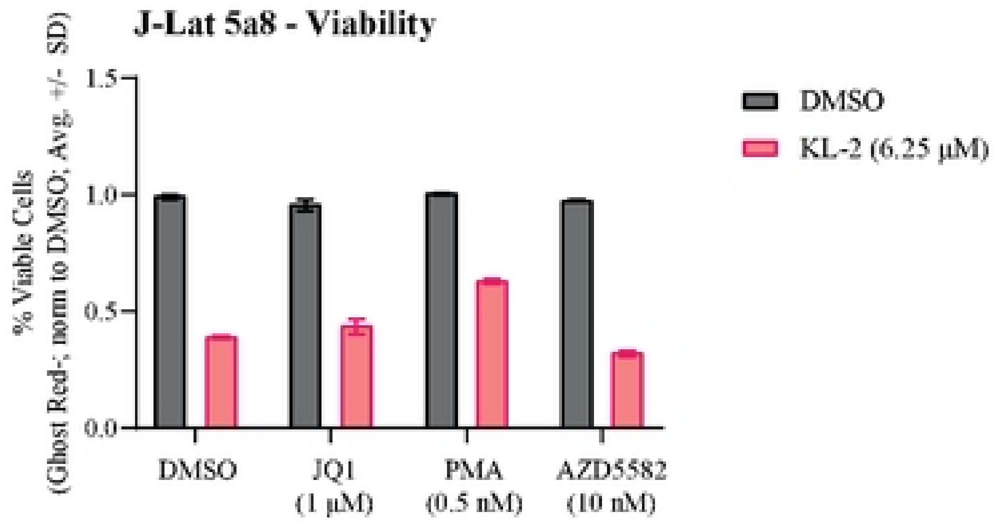

bioRxiv preprint doi: https://doi.org/10.1101/2024.03.01.582881; this version posted March 2, 2024. The copyright holder for this preprint
(which was not certified by peer review) is the author/funder, who has granted bioRxiv a license to display the preprint in perpetuity. It is made
available under aCC-BY 4.0 International license.

B)

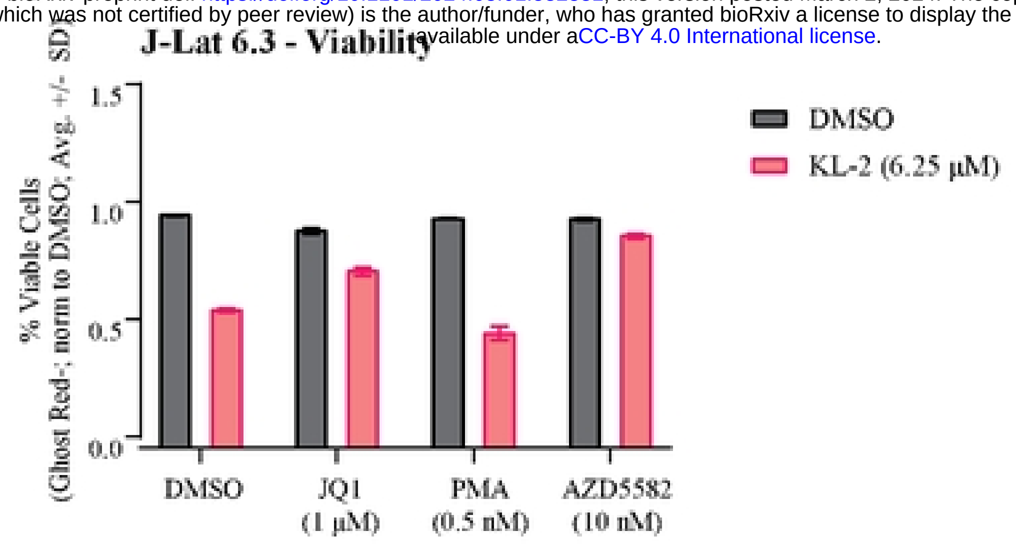

C)

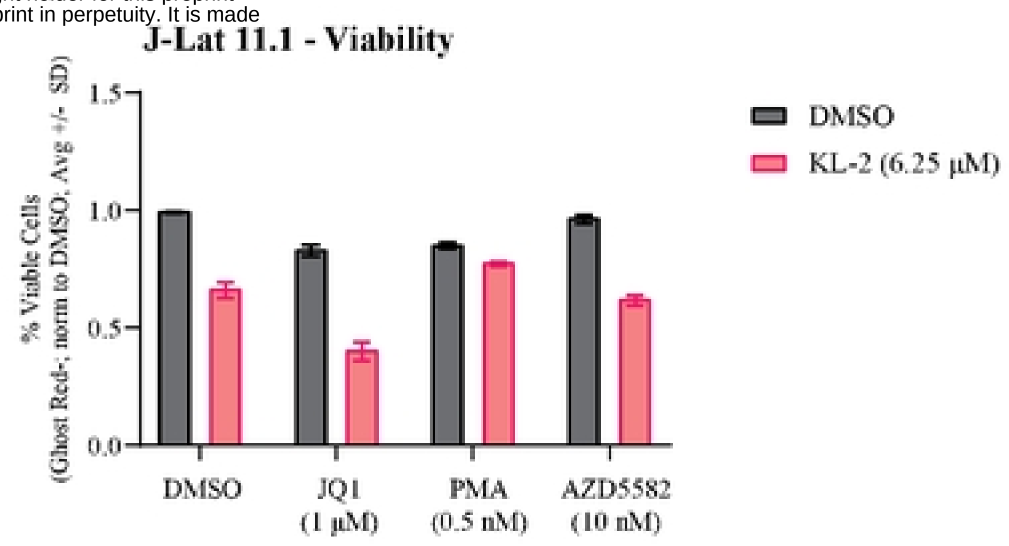

D)

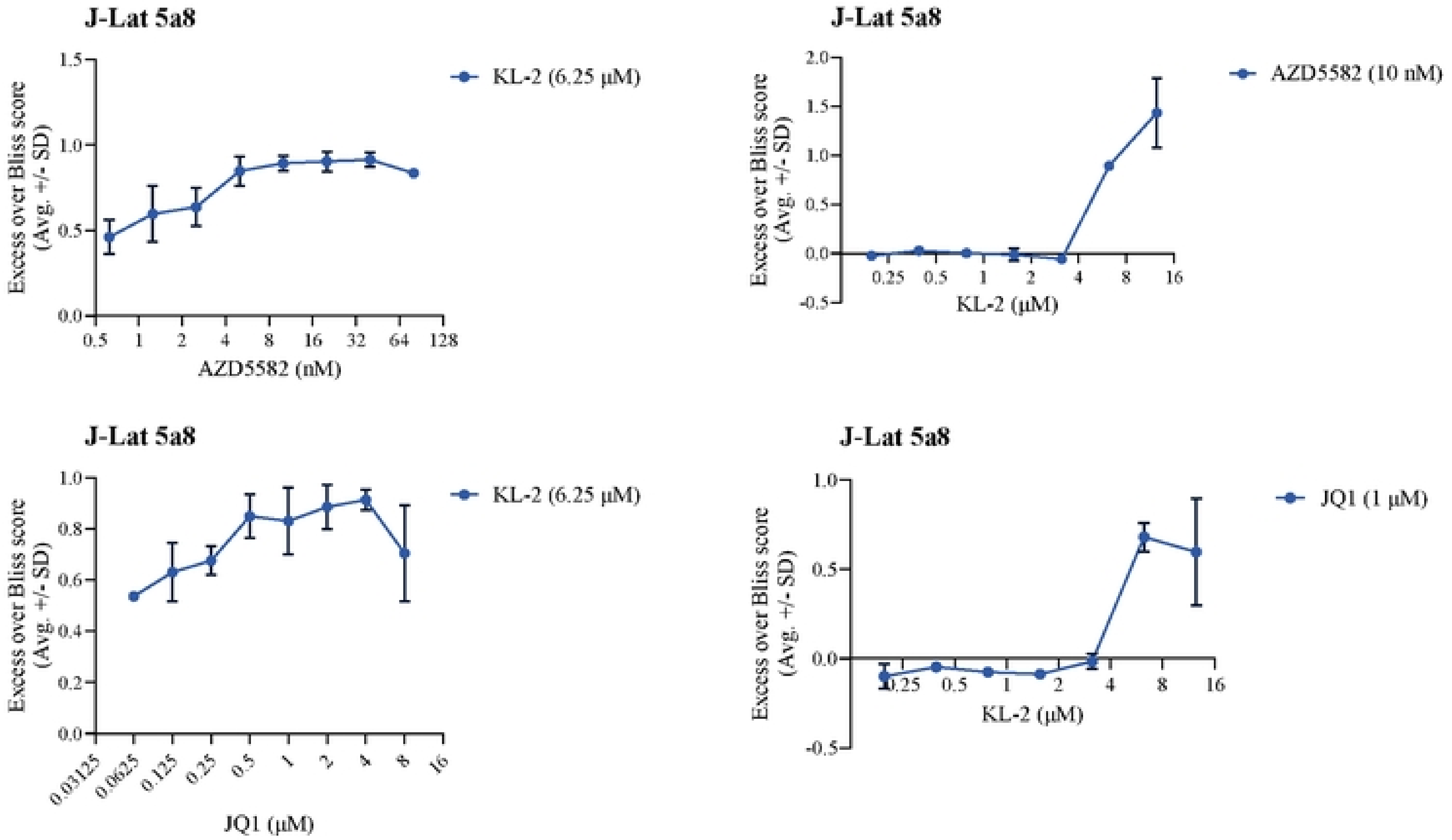

Supplemental Figure 4

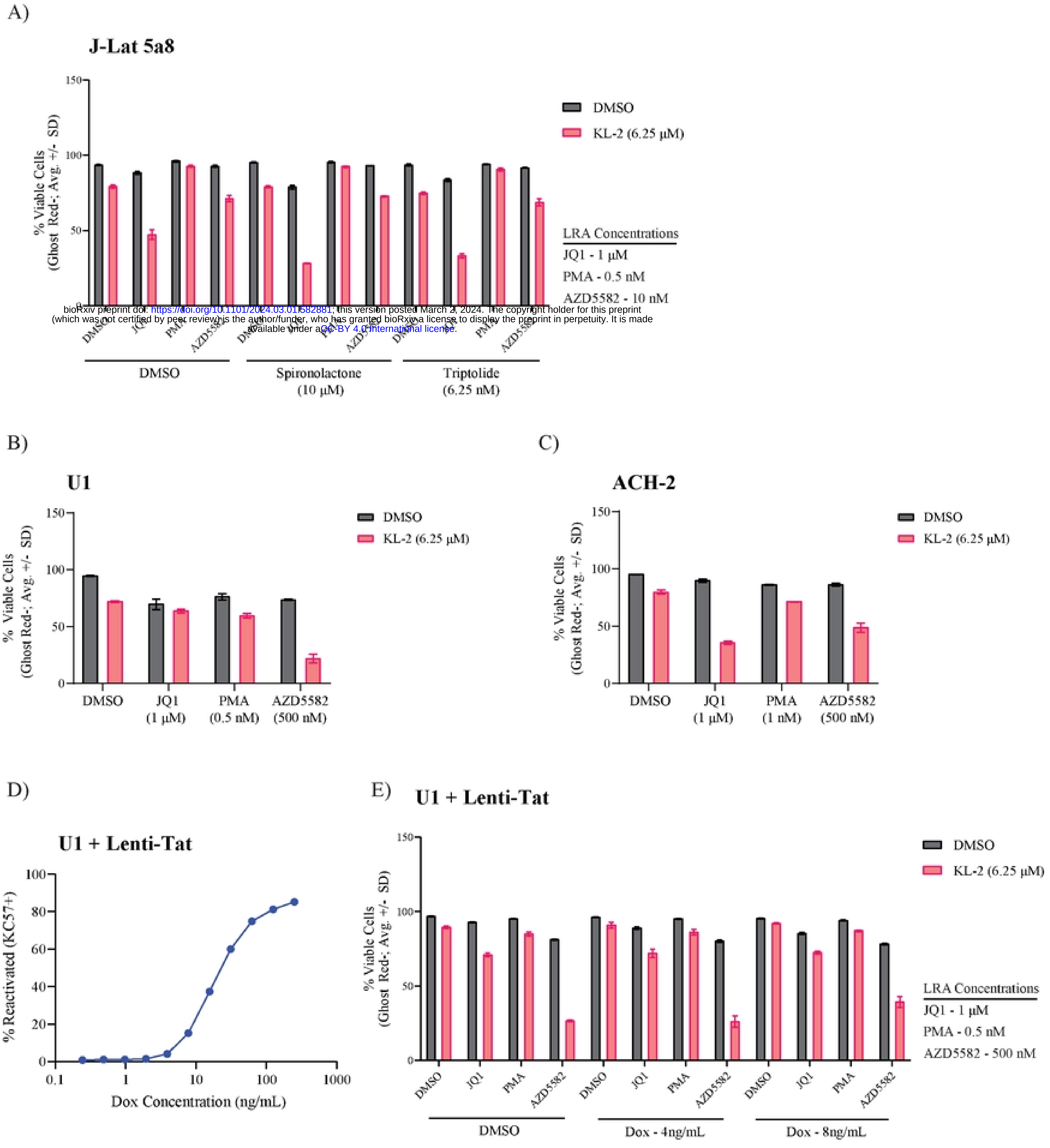

bioRxiv preprint doi: https://doi.org/10.1101/2024.03.01.582881; this version posted March 2, 2024. The copyright holder for this preprint (which was not certified by peer review) is the author/funder, who has granted bioRxiv a license to display the preprint in perpetuity. It is made available under aCC-BY 4.0 International license.
